# On-chip photonics and optoelectronics with a van der Waals material dielectric platform†

**DOI:** 10.1039/d2nr01042a

**Published:** 2022-05-30

**Authors:** Xiaoqi Cui, Mingde Du, Susobhan Das, Hoon Hahn Yoon, Vincent Yves Pelgrin, Diao Li, Zhipei Sun

**Affiliations:** Department of Electronics and Nanoengineering, Aalto University Espoo FI-02150 Finland zhipei.sun@aalto.fi xiaoqi.cui@aalto.fi; QTF Centre of Excellence, Department of Applied Physics, Aalto University Espoo FI-00076 Finland; Université Paris-Saclay, CNRS, Centre de Nanosciences et de Nanotechnologies 91120 Palaiseau France

## Abstract

During the last few decades, photonic integrated circuits have increased dramatically, facilitating many high-performance applications, such as on-chip sensing, data processing, and inter-chip communications. The currently dominating material platforms (*i.e.*, silicon, silicon nitride, lithium niobate, and indium phosphide), which have exhibited great application successes, however, suffer from their own disadvantages, such as the indirect bandgap of silicon for efficient light emission, and the compatibility challenges of indium phosphide with the silicon industry. Here, we report a new dielectric platform using nanostructured bulk van der Waals materials. On-chip light propagation, emission, and detection are demonstrated by taking advantage of different van der Waals materials. Low-loss passive waveguides with MoS_2_ and on-chip light sources and photodetectors with InSe have been realised. Our proof-of-concept demonstration of passive and active on-chip photonic components endorses van der Waals materials for offering a new dielectric platform with a large material-selection degree of freedom and unique properties toward close-to-atomic scale manufacture of on-chip photonic and optoelectronic devices.

## Abstract 2

On-chip dielectric platform using van der Waals materials is experimentally demonstrated for light propagation, emission, and detection, indicating its great potential for faster, smaller, and more efficient photonic integrated circuits.

## Graphical abstract

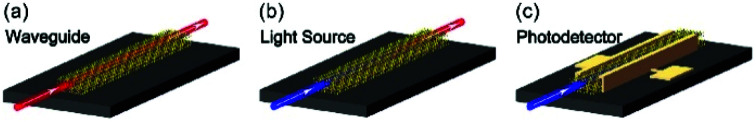

## 1. Introduction

Photonic integrated circuits (PICs) have attracted considerable research interest during the last few decades owing to their broad bandwidth, high operation speed, and power efficiency, promising for various applications, such as data processing,^1–5^ sensing,^6,7^ and inter-chip communications.^8–10^ However, to achieve such a heterogeneous photonic system, light sources, optical modulators, and photodetectors need to be ideally integrated onto the same chip, which remains a formidable challenge for the existing material platforms, including silicon on insulator (SOI),^11,12^ silicon nitride (Si_3_N_4_),^13,14^ indium phosphide (InP),^15,16^ lithium niobate on insulator (LNOI),^17,18^*etc*. For example, SOI and Si_3_N_4_ are the most developed silicon-based material platforms for PICs. Particularly, SOI has achieved great commercial success mainly due to its high index contrast, large third-order nonlinearity, broad transparency window, and compatibility with complementary metal–oxide–semiconductor (CMOS) technology.^11,19^ Nevertheless, the indirect bandgap of silicon/Si_3_N_4_ remains the bottleneck of monolithic light sources and strongly limits the performance of other key active components such as modulators and photodetectors.^20,21^ Moreover, the hybrid integration of silicon and III/V materials or germanium is also challenging because of lattice mismatch and thermal expansion issues.^9,22^ Apart from silicon, InP is another widely explored photonic platform that exhibits excellent performance in active applications,^15,16^ but InP is not compatible with CMOS technology, which has become the obstacle that prevents further commercialisation.^23^ LNOI is a revolutionary material platform, possessing attractive material properties (*e.g.*, an ultrabroad low-loss transparency window, high second-order optical nonlinearity, and high electro-optical coefficient)^24^ that have led to various competitive on-chip devices (*e.g.*, optical modulators^18,25^). However, LNOI suffers from its subtle index contrast,^26^ which results in a larger footprint and decreases the integration density.^18,27^

**Fig. 1:**
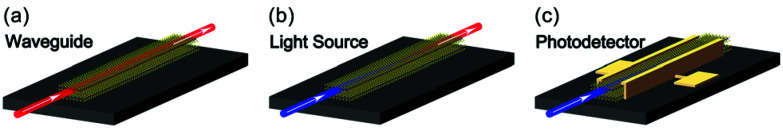
The concept of the on-chip vdW material dielectric platform. The scheme of the building blocks with such a platform, in which vdW materials serve as a passive and active dielectric medium for (a) light-guiding waveguides, (b) on-chip light sources, and (c) on-chip integrated photodetectors. In (b), the waveguide emits light (red beam) after absorbing the pump light (blue beam). In (c), the electrodes on both sides of the waveguide represent the source and drain that collect the photocurrent generated by the incident light (blue beam) in the waveguide.

Recently, the concept of all van der Waals (vdW) material^28–37^ integrated nanophotonics has been put forward by flawless simulation,^38^ in which vdW materials function as a dielectric medium, guiding, converting, and detecting light that propagates inside, as shown in Fig. 1. Similar to silicon, vdW materials exhibit a naturally high refractive index in the near-infrared range. For example, the refractive index along basal planes ranges from ∼3.9 for WS_2_ and WSe_2_ to ∼4.4 for MoS_2_ and MoSe_2_,^36,39^ which provides an ultrabroad transparency window^40,41^ with a much higher confinement factor^38^ for the light with photon energy smaller than the bandgap (*i.e.* the bandgap of bulk MoS_2_ is ∼1 eV,^42^ corresponding to a transparency window of wavelength ≳1.2 μm). Furthermore, the additional simulation shows that the strong light confinement in vdW materials leads to an enhanced light–matter interaction.^38^ The effective mode volume in the MoS_2_ cavity is 45% smaller, and the Purcell enhancement is four times higher than that of silicon and GaAs.^38^ Besides, a wide range of vdW materials have been discovered and developed during the last few decades, including insulators (*i.e.*, h-BN^43,44^), semiconductors (*i.e.*, black phosphorus^45,46^ and transition metal dichalcogenides^30^), conductors (*i.e.*, graphene^28^ and MXenes^47,48^) and superconductors (*i.e.*, NbSe^49^), which provides great flexibility in material selection and a possibility for the design and engineering of various passive and active photonic and optoelectronic devices.

In this work, we experimentally demonstrate the concept of an on-chip vdW material dielectric platform, in which nanostructured bulk vdW materials function as a passive and active dielectric medium for on-chip light-guiding waveguides, light sources, and integrated photodetectors. As a proof of concept, our results advance the merits of vdW materials, such as ultra-flat surfaces and high flexibility in material selection and band engineering, which make vdW materials a new dielectric platform that holds great potential for on-chip photonics and optoelectronics.

## 2. Light guiding in vdW material waveguides

**Fig. 2:**
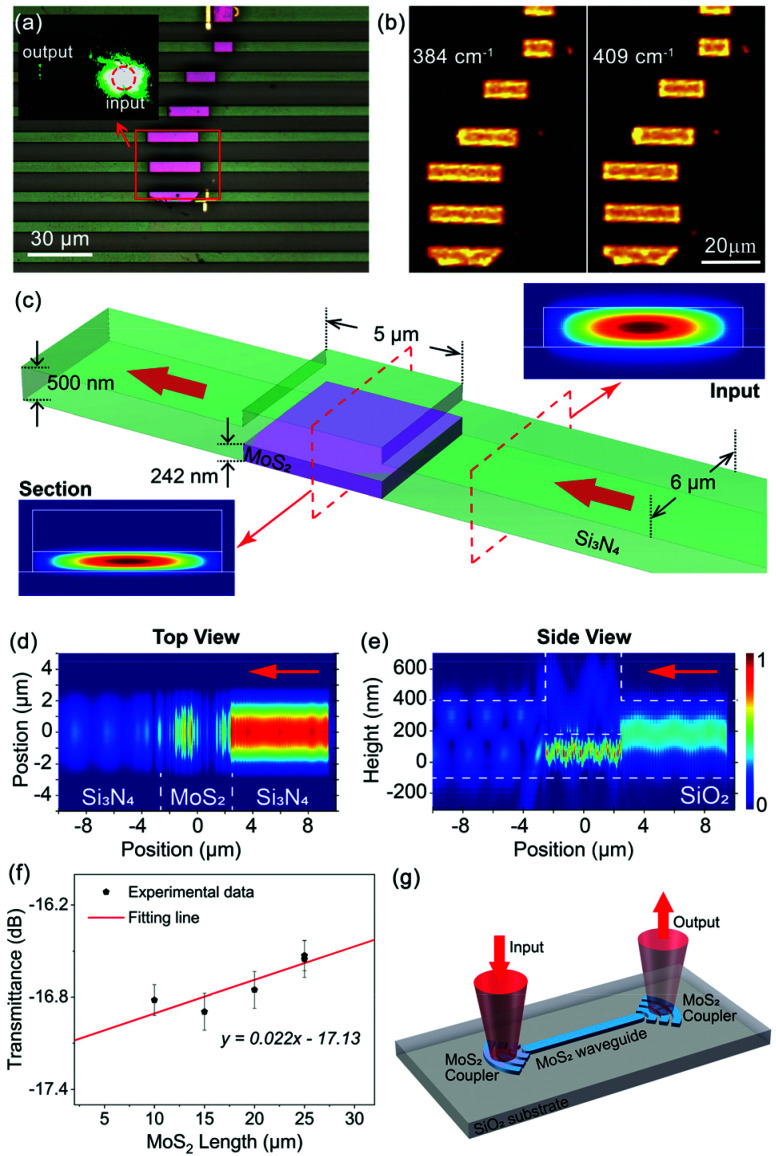
Demonstration of light-guiding in vdW material waveguides. (a) A microscopy image of the integrated MoS_2_ waveguides, in which the light green parts are the Si_3_N_4_ waveguides and the light purple parts are the MoS_2_ waveguides; inset: an image of light coupling and propagating inside the MoS_2_ waveguide; (b) Raman mapping images created by collecting the Raman intensity at the central wavenumbers of 384 cm^−1^ (left) and 409 cm^−1^ (right) in a range of 10 cm^−1^ separately; (c) the schematic applied in the simulation and the simulation result of the optical modes regarding the cross-sections of the Si_3_N_4_ waveguide and MoS_2_ waveguide; and revolution of the incident light from a top view (d) and a side view (e). The red arrows indicate the direction of the incident light; (f) the measured transmittance of the integrated waveguides *versus* the length of MoS_2_; and (g) the design of a vdW material-based waveguide with grating couplers. The experimental data are collected at a wavelength of 632 nm.

To be an effective material platform for PICs, various characteristics are needed, such as a wide transparency range, high index contrast, and flexibility in manufacturing, which endow it with the capacity to provide low-loss and compact components for high-performance active and passive applications. Among all the building blocks, a low-loss waveguide is the most significant component. Here, we demonstrate MoS_2_ waveguides (details in the Device fabrication section) to prove the light-guiding concept in vdW material waveguides. Fig. 2(a) shows the microscopy image of various bulk MoS_2_ waveguides, with the MoS_2_ length ranging from ∼10 to 25 μm. In addition, the MoS_2_ waveguides are further integrated into Si_3_N_4_ waveguides (light green strips in Fig. 2(a), the scheme is in ESI Fig. S1a†) separately for light coupling. Besides, the Raman spectrum is shown in ESI Fig. S2,† and Raman mappings regarding the two distinct peaks at ∼384 cm^−1^ and ∼409 cm^−1^ are shown in Fig. 2(b), respectively, indicating that the MoS_2_ flake is fully patterned and integrated into Si_3_N_4_ waveguides. The geometry and thickness of the waveguides are characterized by atomic force microscopy (AFM) and presented in ESI Fig. S3,† in which the ultra-uniformity demonstrates the low surface roughness of the waveguides. The ∼242 nm step of the with/without integration area indicates the thickness of the MoS_2_ flake, and the ∼529 nm thickness difference between the waveguide and the substrate additionally proves that the 500 nm Si_3_N_4_ layer is thoroughly etched.

To demonstrate the light propagation inside the MoS_2_ waveguide, a 532 nm laser is directly coupled into the MoS_2_ waveguide *via* a 50× objective (NA = 0.75). The output light is clearly observed at the other end of the waveguide, as indicated by the inset of Fig. 2(a). However, with the direct coupling method, the linear loss cannot be experimentally demonstrated because the optical power that is injected strongly depends on the coupling status and is rarely constant. Therefore, we pattern the MoS_2_ waveguides to induce the same facets and utilise Si_3_N_4_ waveguides to inject and collect the light. As the simulation results demonstrated in Fig. 2c–e, the propagating light in the Si_3_N_4_ waveguide is efficiently injected into the MoS_2_ waveguide, and the output is well collected by the following Si_3_N_4_ waveguide. Subsequently, the loss of the MoS_2_ waveguides is measured using a homemade waveguide coupling system (shown in ESI Fig. S4†) by the ‘cut-back’ method.^50^ The experimental results and the linear fitting line are plotted in Fig. 2(f). According to the fitting result, the insertion loss of the whole waveguides is ∼17.13 dB, which is relatively high compared with the pure Si_3_N_4_ waveguides (the results are plotted in ESI Fig. S5†) and previous works.^50–52^ However, the linear loss of the MoS_2_ waveguide part is calculated to be −0.02 dB μm^−1^, which is negligible. We attribute this to the ultra-smooth surface of the MoS_2_ flake compared with the Si_3_N_4_ film grown by plasma-enhanced chemical vapor deposition (PECVD). As illustrated in ESI Fig. S6,† the MoS_2_ waveguides show an average surface roughness of ∼2 nm, which is more than five times lower than that of the Si_3_N_4_ waveguides. Thus, the negative loss is expected as the MoS_2_ waveguides have superior surface and better confinement, which results in lower total loss when longer MoS_2_ waveguides are integrated. Note that, recently, it has been shown that the surface roughness is crucial to reduce the waveguide loss for applications (*i.e.*, frequency combs^53,54^). In this aspect, silicon photonics has enabled great successes, and recently it has been shown that the surface roughness is reduced to a few nanometers for high-quality resonators.^55^ Here, due to the atomic scale nature, the vdW material waveguide concept can reach an ultimate limit in surface quality (*i.e.*, atomically smooth surface) without complex manufacturing like chemical mechanical polishing.^53,55^ Apart from light injection *via* Si_3_N_4_ waveguides, a grating structure that is fabricated from vdW materials can also be considered, which is suitable for achieving light coupling in a limited region, as demonstrated in Fig. 2g.

## 3. On-chip light sources with vdW materials

**Fig. 3:**
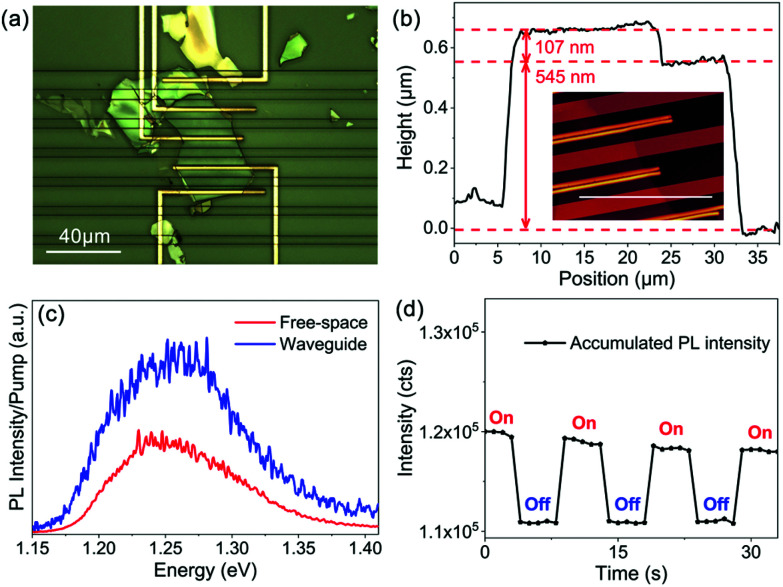
Demonstration of on-chip optically pumped light sources with vdW materials. (a) A microscopy image of the InSe integrated devices. Note that the contacts fabricated here are not relevant to the optically pumped light sources; (b) the section height corresponding to the profile indicated by the white line in the image of AFM characterisation (inset); (c) PL efficiency collected *via* free-space pumping (red) and waveguide pumping (blue); and (d) excitation-controlled accumulated PL intensity with an on/off period of ∼10 seconds.

The vdW material family has grown dramatically during the last few decades, and various vdW materials have been discovered and demonstrated, ranging from insulators (h-BN), semiconductors (transition metal dichalcogenides) to conductors (MXenes), which offers great flexibility for the proposed vdW material dielectric platform. As for the concept demonstration of on-chip light sources, the suitable candidate should allow a strong light–matter interaction, which brings two requirements of strong light absorption and a direct bandgap. Therefore, here we select InSe^56–58^ as an example for the demonstration of on-chip light sources with the vdW material dielectric platform (details in the Device fabrication section) because bulk InSe possesses not only a suitable band structure for light emission,^59^ but also an out-of-plane absorption dipole which further increases the light–matter interaction.^60,61^ As shown in Fig. 3(a), different from the MoS_2_ integrated device, the InSe flake is not fully etched away for the convenience in fabricating the electrodes. The scheme of this device is shown in ESI Fig. S1b.† For characterisation, AFM is first carried out, and the result is shown in Fig. 3(b), in which the thickness of the InSe flake is calculated to be ∼107 nm. The red curve in Fig. 3(c) shows the result of photoluminescence (PL) characterisation of the integrated light source, a strong peak centered at ∼1.25 eV is measured. Raman characterisation is included in ESI Fig. S8.† The results demonstrate the high quality of the flake and well agree with previously published results.^59^

As the blue curve plotted in Fig. 3(c), the PL signal of the on-chip light source (details in Experimental methods) exhibits the same peak of ∼1.25 eV as the PL spectrum collected from the free-space pumping, indicating the existence of light–matter interaction between the propagating modes and the InSe waveguides. The curves in Fig. 3(c) are further normalised by the pump power (details in Experimental methods), according to which the PL efficiency of the waveguide pump is ∼1.86 folds higher than that of the free-space pump. We attribute this enhancement to the effective absorption caused by the waveguide structure and the out-of-plane dipole of InSe.^60^ Furthermore, an excitation-controlled PL measurement is implemented by periodically switching on/off the pump laser with a mechanic shutter, and the data are presented in Fig. 3(d). Clearly, the accumulated PL created by summing the PL intensity from the energy of ∼1.22 eV to 1.32 eV exhibits the same on/off state simultaneously with the pump laser. So far, although the performance is not studied in detail, on-chip controllable light sources are achieved in the InSe integrated waveguides to prove the concept. Here, it is worth mentioning that electrically pumped light sources, which are of great interest in practical applications, are achievable in this geometry with suitable electrodes.

## 4. On-chip photodetectors with vdW materials

**Fig. 4:**
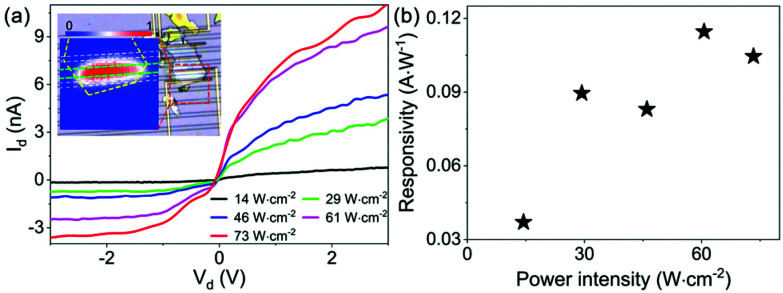
Demonstration of on-chip photodetectors with vdW materials. (a) *I*_d_–*V*_d_ curves collected from the InSe waveguide with different power intensities at ∼520 nm, inset: photocurrent mapping result, in which the dashed lines show the silhouette of the device, and the mapping area is indicated by the red dashed rectangular and (b) calculated photo-responsivity *versus* power intensity in the case of *V*_g_ = −60 V and *V*_d_ = 2 V.

Integrated photodetectors are the vital active components for PICs. Here, we demonstrate the applicability of on-chip active optoelectronic components in the vdW material dielectric platform using the same InSe integrated device with metallic contacts (shown in Fig. 3a, details in the Device fabrication section). Firstly, a photocurrent mapping (details in the Experimental method section) is implemented to characterise the workability of the devices. As illustrated in the inset of Fig. 4(a), the whole flake area between the metal electrodes shows photoresponse, and a clear hot area is observed below the waveguide part, indicating that the integrated photodetector is fully functional.

Subsequently, the devices are measured *via* waveguide coupling (details in the Experimental methods section), and *I*_d_–*V*_d_ curves are collected at different incident intensities with the same gate voltage of −60 V, as plotted in Fig. 4(a). The drain current *I*_d_ clearly depends on the power intensity, but the *I*_d_–*V*_d_ curves exhibit an apparent rectifying effect. This is due to the Schottky barrier at the surface of the flake and the Ti/Au electrodes, which is also confirmed in the excitation-controlled measurement (results are shown in ESI Fig. S9†). The photo-responsivity *versus* the incident intensity is calculated according to the data measured in the case of *V*_g_ = −60 V and *V*_d_ = 2 V and shown in Fig. 4(b). Based on the result, the highest photo-responsivity is ∼0.115 A W^−1^ at the intensity of ∼60 W cm^−2^. As a comparison, the devices are demonstrated *via* free-space coupling, and the results are shown in ESI Fig. S10.† The photo-responsivity of the on-chip integrated photodetector is two times higher than that of the free-space measurement. The enhancement of the photo-responsivity originates from the higher light absorption resulted from the resonance between the out-of-plane dipole and the propagating modes, which is entirely excluded in the case of free-space coupling. Nonetheless, the photo-responsivity is relatively low compared with other reports,^62–64^ but it can be much improved by optimising the fabrication processes and the design of the device, *e.g.*, introducing an oxygen- and moisture-free environment by adding h-BN^65^ or reducing the carrier scattering at the oxidised interface by changing to polymethyl methacrylate (PMMA) substrates.^58,66^ Nevertheless, here, we demonstrate the concept of on-chip photodetectors with the vdW material dielectric platform.

## 5. Conclusions

Here, we have demonstrated three vital building blocks of PICs with the concept of the vdW material dielectric platform, including light-guiding waveguides, on-chip light sources, and photodetectors. Note that optical modulators,^30^ nonlinear^67^ optical frequency converters, and high-quality cavities^36^ are possible with the vdW material platform. Compared with the currently dominating material platforms (such as SOI, Si_3_N_4_, InP, and LNOI), the vdW material dielectric platform exhibits various unique and complementary advantages, *i.e.*, an inherent ultra-flat surface, selection of various materials as well as the CMOS compatibility. These merits offer great freedom in manufacturing, band engineering, and heterogeneous integration. However, it is worth mentioning that the challenge remains in obtaining environmentally stable, large-scale, and high-quality vdW materials, although the rapidly growing community has recently demonstrated wafer-size single-crystal semiconducting vdW materials.^68–70^ In summary, our results show that the vdW material dielectric platform holds great potential in PICs for future on-chip photonics and optoelectronics.

## 6. Device fabrication

### Device substrate

SiO_2_ is deposited (PECVD, Oxford Plasmalab 80Plus, ∼1720 nm) onto a heavily p-doped Si wafer which has original the ∼280 nm thermal oxidised SiO_2_ on the surface.

### MoS_2_ integrated device

The mechanically exfoliated MoS_2_ (purchased from 2D Semiconductors) flake is transferred onto the substrate. Afterward, Si_3_N_4_ (500 nm) is deposited on top of the chip by PECVD. Subsequently, the PMMA (A9) photoresist is spin-coated and patterned *via* the standard e-beam lithography (EBL, Vistec EBPG 5000) method. Finally, the waveguide structure is fabricated by dry etching and plasma-enhanced reactive ion etching (RIE Plasma, Oxford Plasmalab 80Plus) using CF_4_ gas. The sample is cleaved by a commercial dicing tool (Disco DAD 3220).

### InSe integrated device

InSe (purchased from 2D Semiconductors) is mechanically exfoliated and transferred onto the substrate. After that, Si_3_N_4_ (500 nm) is deposited onto the surface by PECVD. The waveguides are fabricated by the standard EBL process and RIE Plasma dry etching using CF_4_ gas. Afterward, the Ti/Au electrodes are fabricated by the standard EBL process and angled metal deposition (MASA IM-9912) with −30°, 0°, and 30° separately followed by a lift-off process. Then, a protecting SiO_2_ (120 nm) layer is deposited on the top by PECVD. Finally, the chip is cleaved using a commercial dicing tool (Disco DAD 3220).

## 7. Experimental methods

### Characterisation methods

Raman analysis is conducted using a micro-Raman system (WITec alpha300) with a 532 nm laser; AFM images are obtained using a Dimension Icon system (Bruker); PL images and spectra are collected with a WITec alpha300 system using a 20× objective (NA = 0.4) and a 532 nm laser as the excitation source. Data are further normalised by the intensity of the pump and the NA is taken into consideration.

### PL measurement *via* waveguide coupling

The sample is placed onto a homemade three-dimensional alignment stage, which allows light injection from a tapered fibre to the waveguide. The whole alignment stage is then placed in the WITec alpha300 system for data collection. A 532 nm laser with an intensity of ∼400 W cm^−2^ is coupled into the waveguide as the pump, and the PL signal is collected by a 50× objective (NA = 0.75). Data are further normalised by the intensity of the pump and the NA is taken into consideration.

### Photocurrent mapping

Photocurrent mapping is performed using the WITec alpha300 system with light coupling from the top of the devices *via* a 20× objective (NA = 0.4). The sample is connected to a print circuit board for data collection. Keithley 2401 and Keithley 2400 are used for applying the source/drain and gate. Two source meter units and the WITec alpha300 system are precisely controlled by a homemade LabVIEW software, which allows synchronised data collection with the moving stage. The laser used in the experiment is 50 μW/532 nm.

### Optoelectrical measurement *via* waveguide coupling

The measurement is carried out using a homemade waveguide coupling system (illustrated in ESI Fig. S4†). Light is coupled into the waveguide *via* a tapered fibre, and Keithley 2401 and Keithley 2400 are used for applying the source/drain and gate. The two source meter units are grounded and controlled by a homemade LabVIEW software for data collection.

All measurements are performed in an ambient environment.

## Author contributions

Z. S. conceived the project. X. C. fabricated the MoS_2_ integrated and InSe integrated devices and characterised the devices with the help of M. D., S. D., H. H. Y., D. L. and X. C. M. D., S. D., and H. H. Y. carried out the photonic and optoelectronic measurements. V. Y. P. carried out the numerical simulation. X. C. and Z. S. analysed the data and wrote the manuscript with input from all authors. All authors reviewed the manuscript.

## Conflicts of interest

There are no conflicts to declare.

## Supplementary Material

NR-014-D2NR01042A-s001
