# Supplementary material for: On-chip photonics and optoelectronics with a van der Waals material dielectric platform
Source: Nanoscale. 2022 May 30;14(26):9459–65. doi: 10.1039/d2nr01042a (PMC9261272; doi:10.1039/d2nr01042a)
Supplement: NR-014-D2NR01042A-s001 [file NR-014-D2NR01042A-s001.pdf]

Electronic Supplementary Material (ESI) for Nanoscale.
This journal is © The Royal Society of Chemistry 2022

Supplementary Information

On-chip photonics and optoelectronics with van der Waals material dielectric platform

Xiaoqi Cui, *[a,b] Mingde Du, [a] Susobhan Das, [a] Hoon Hahn Yoon, [a,b] Vincent Yves Pelgrin, [a,c] Diao Li [a,b] and Zhipei Sun *[a,b]

[a] *Department of Electronics and Nanoengineering, Aalto University, Espoo FI-02150, Finland*

[b] *QTF Centre of Excellence, Department of Applied Physics, Aalto University, Espoo FI-00076, Finland*

[c] *Université Paris-Saclay, CNRS, Centre de Nanosciences et de Nanotechnologies, 91120, Palaiseau, France*

Email: zhipei.sun@aalto.fi, xiaoqi.cui@aalto.fi

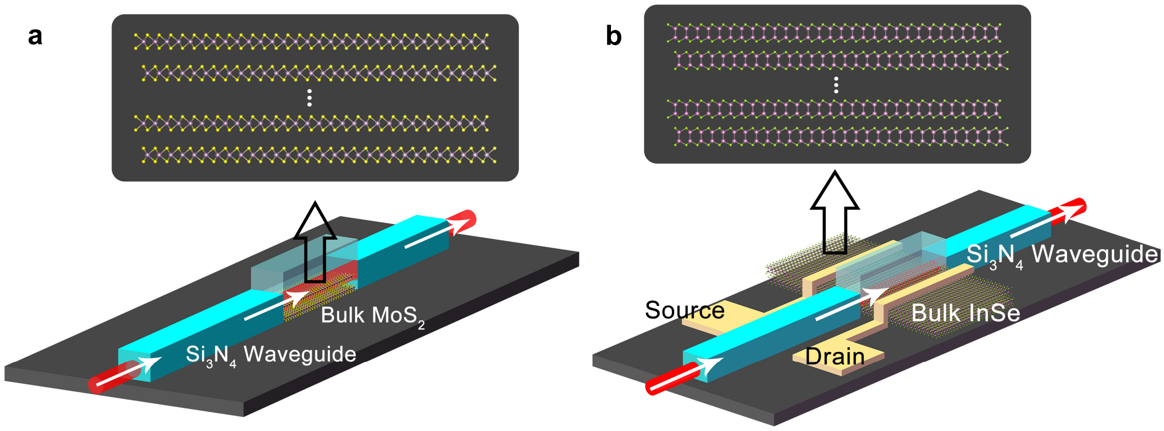

Figure S1, The schemes of the MoS_2 integrated and InSe integrated devices. a is the scheme of the MoS_2 integrated Si_3N_4 waveguide, in which the Si_3N_4 waveguide with a dimension of 6 μm width and 500 nm height is cladded by the SiO_2 at the bottom and the air at the sides and top. At the same time, the ~242 nm thick MoS_2 waveguide with an ultra-smooth surface is integrated into the Si_3N_4 waveguide. Note that the width of the waveguide is designed as 6 μm because of the relatively low quality of the Si_3N_4 films grown by the PECVD available in the group. **b** is the scheme of the InSe

integrated Si_3N_4 waveguide, in which the width of the waveguide is also designed as 6 μm as in this case the waveguide allows multimode propagation with an acceptable linear loss (~1.79 dB·mm^{-1} at 532 nm, as shown in Figure S7, the pure Si_3N_4 waveguides are fabricated along with the InSe integrated ones following the same parameters).

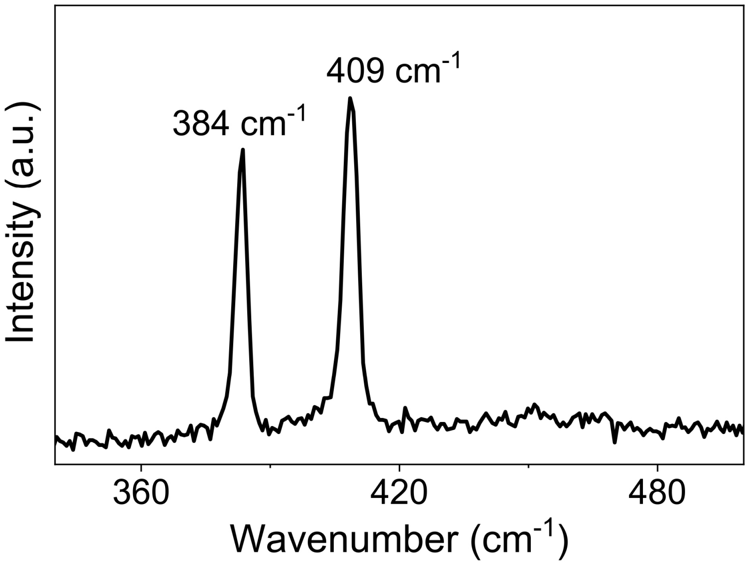

Figure S2, Raman spectrum of the MoS_2 waveguides.

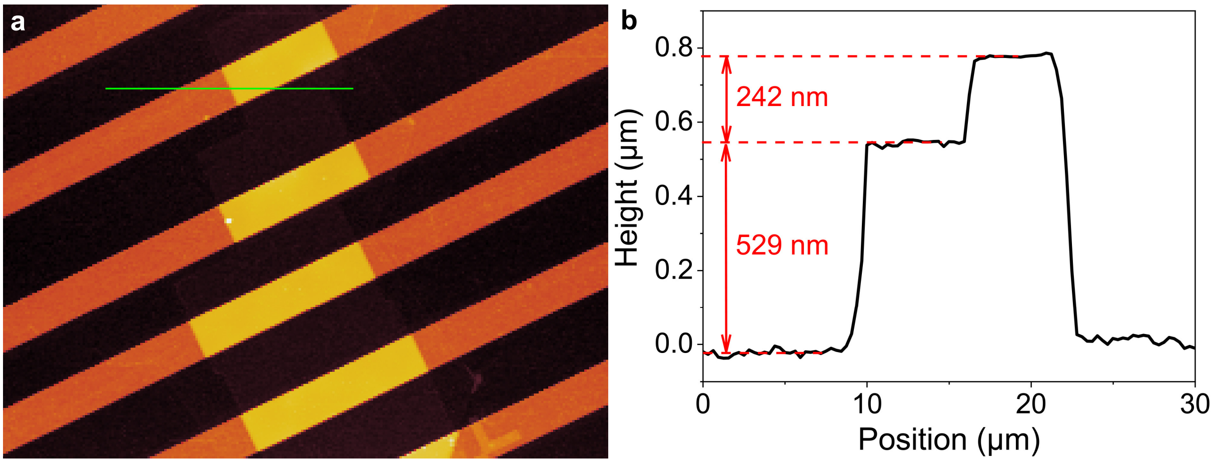

Figure S3, AFM characterization of the MoS_2 integrated devices. **a** The AFM image and **b** the height profile corresponding to the section (indicated by the green line in **a**).

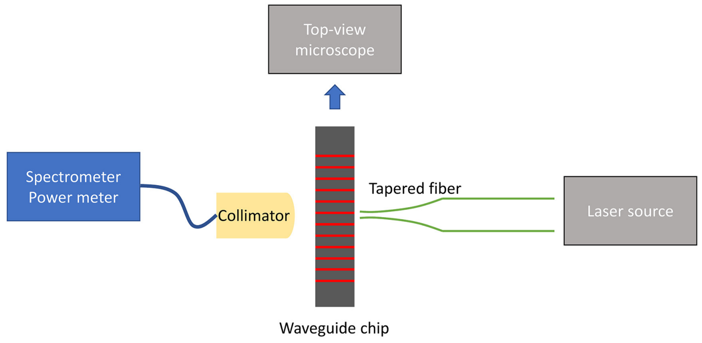

Figure S4, Scheme of the homemade waveguide coupling system.

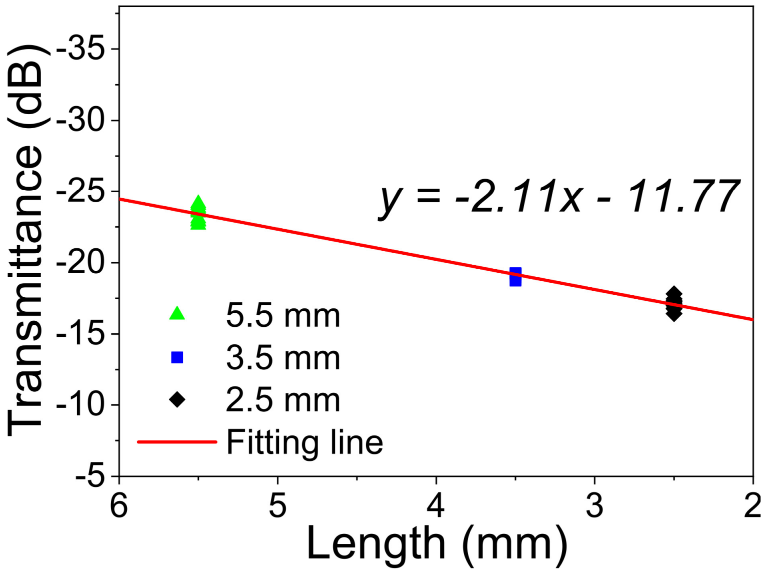

Figure S5, Loss measurement results and linear fitting line of the pure Si_3N_4 waveguides, which are fabricated together with the MoS_2 integrated ones following the same parameters. The insertion loss and the linear loss of the Si_3N_4 waveguide are 11.77 dB and 2.11 dB, respectively. The measurement is carried out by the cut-back method at 632 nm.

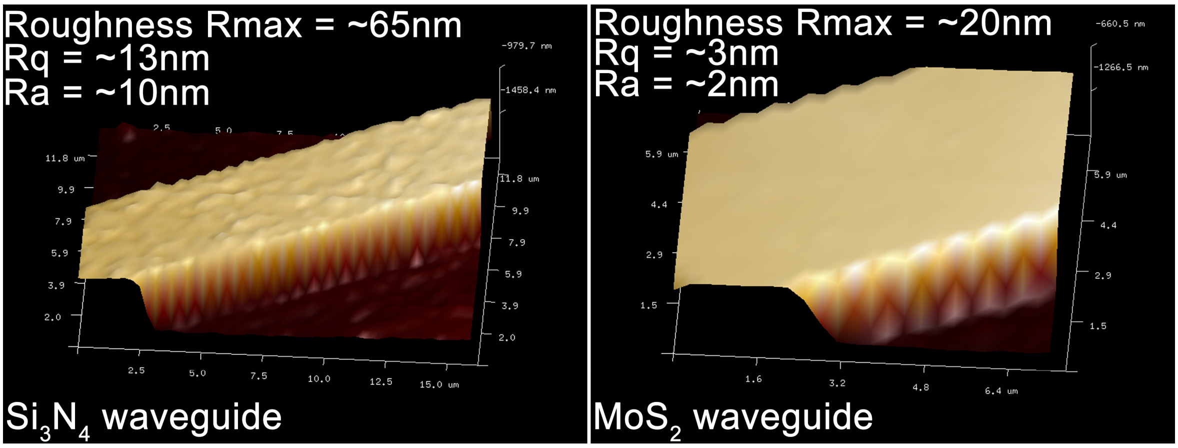

Figure S6, Three-dimensional graphs of Si_3N_4 waveguide and MoS_2 waveguide measured by AFM. The waveguides are fabricated together following the same parameters. The calculated roughness R-max= ~65 nm, Rq= ~13 nm, Ra= ~10 nm for Si_3N_4 waveguides and R-max= ~20 nm, Rq= ~3 nm, Ra= ~2 nm for MoS_2 waveguides, respectively.

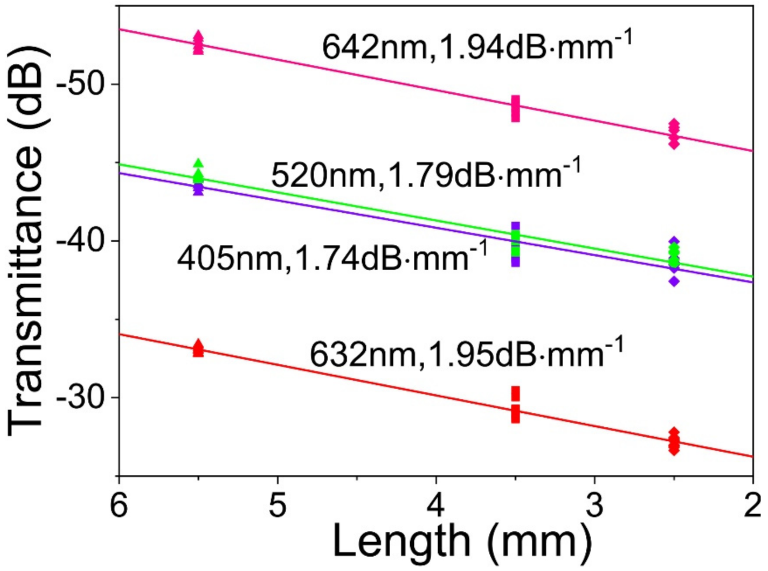

Figure S7, Linear loss of Si_3N_4 waveguides measured from the comparison sample which is fabricated along with the InSe integrated ones following the same parameters. The measurement is carried out by cut-back method.

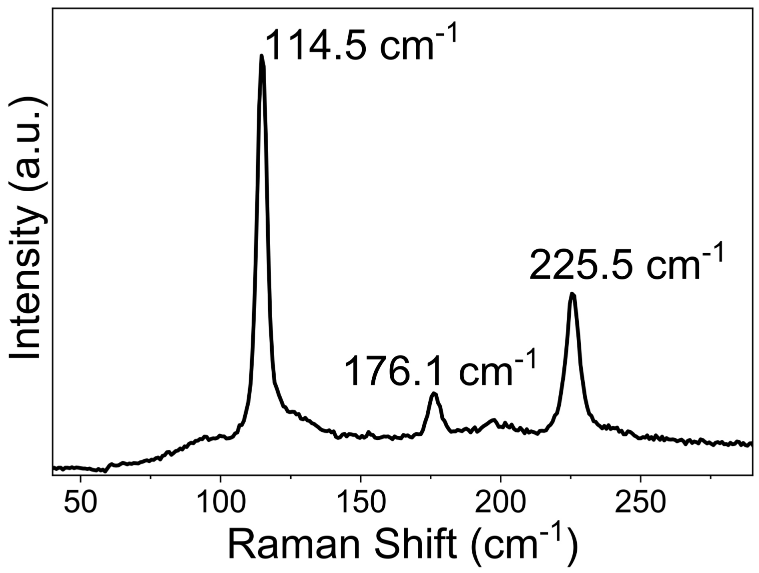

Figure S8, Raman spectrum of the InSe flake. The distinct InSe peaks at ~115 cm^{-1}, ~176 cm^{-1}, and ~226 cm^{-1}, corresponding to A_g, E^2_g, and A^2_g phonon modes, respectively.

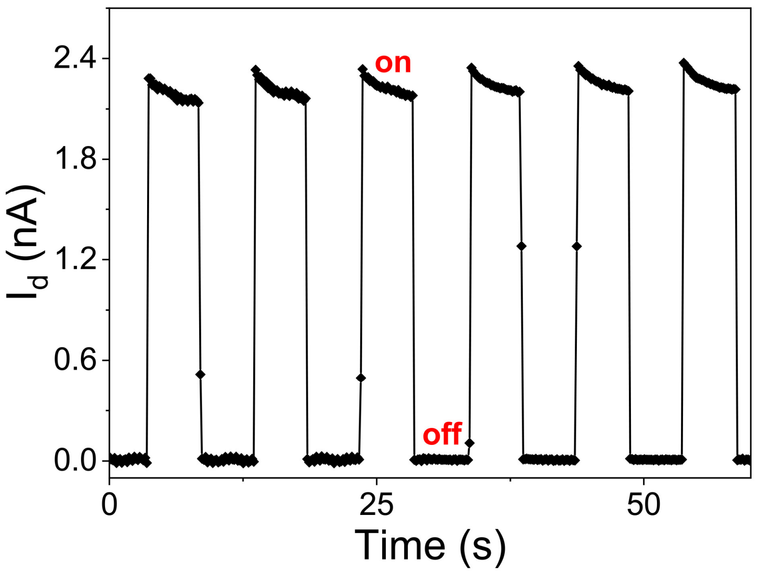

Figure S9, Excitation-controlled measurement with 532 nm pump laser which is controlled in a 10-second on/off period. The measurement is carried out using free-space coupling. From the diagram, one can clearly observe periodic on and off states of the drain current I_d, which corresponds to the laser on and off, respectively.

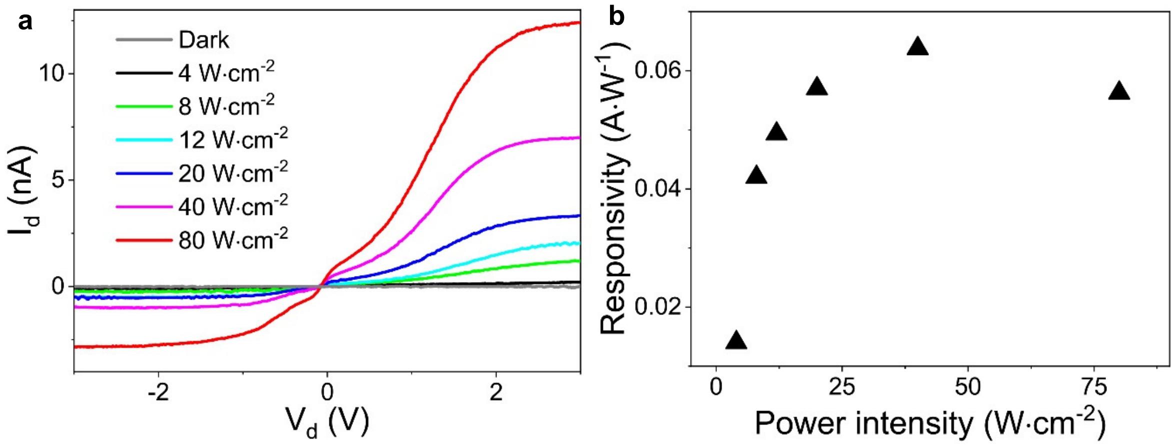

Figure S10, Demonstration of on-chip photodetectors with vdW materials. a I_d-V_d curves of free-space coupling with 532 nm laser illumination, Vg=-60 V; **b** the calculated photo-responsivity of free-space coupling at different incident intensity, V_g=-60 V and V_d=2 V. The free-space measurement is done in the WITec alpha300 system with a 20x objective (NA=0.4), the sample is connected to a print circuit board. Keithley 2401 and Keithley 2400 are used for applying source/drain and gate. The two source meter units are grounded and controlled by a homemade LabVIEW software for data collection.

According to the photocurrent mapping result (shown in the inset of Fig. 4**a**), the laser beam is focused on the highest photo-response area for the measurement. As shown in Fig. S10**a**, I_d-V_d curves are collected at different incident intensity under the same gate voltage of -60V. When the laser illuminates on the flake, the drain current I_d shows clear dependence on the intensity, but the I_d-V_d curves exhibit an apparent rectifying effect. This is due to the Schottky barrier at the surface of the flake and the Ti/Au electrodes. The photo-responsivity is calculated in the case of V_g=-60 V and V_d=2 V and plotted in Fig. S10**b**, in which the highest photo-responsivity is 0.06 $A{\cdot}W^{-1}$ at the intensity of ~40 $W{\cdot}cm^{-2}$.
